# Furosemide and albumin for diuresis of edema (FADE): a study protocol for a randomized controlled trial

**DOI:** 10.1186/1745-6215-15-222

**Published:** 2014-06-12

**Authors:** Simon JW Oczkowski, Ian Mazzetti, Maureen O Meade, Cindy Hamielec

**Affiliations:** 1 Division of Critical Care Medicine, McMaster University, Hamilton, ON, Canada; 2 Critical Care Medicine Residency Program, Room 2U c/o Anesthesia Department, McMaster University, 1200 Main St. W., Hamilton, ON L8N 3Z5, Canada

**Keywords:** Albumin, Critical care, Edema, Furosemide, Diuresis, Post-resuscitation

## Abstract

**Background:**

Fluid retention is a common complication of critical illness. It typically results from large-volume fluid infusions during acute resuscitation and is worsened by hypoalbuminemia. Recognized as edema, fluid retention is important for its association with delayed weaning and increased mortality. The standard treatment is the administration of diuretics, with or without albumin. We hypothesize that intravenous 25% albumin plus furosemide, by comparison with furosemide alone, improves diuresis, oxygenation, and hemodynamic stability in the deresuscitation of critically ill, hypoalbuminemic patients. We propose a pilot study to determine the feasibility of a trial to investigate this hypothesis.

**Methods/Design:**

FADE is a single-center, parallel, pilot randomized controlled trial. We aim to allocate 50 hemodynamically stable, hypoalbuminemic adult patients receiving diuresis to treatment with either 100 ml of either 25% albumin or normal saline placebo twice daily, for a total of six doses. Diuretics are to be prescribed by the caregiving team at least twice daily, and administered within 2 hours following study treatment. Patients, intensive care unit (ICU) clinicians, data collectors, and outcome adjudicators will be blinded to treatment allocation. Feasibility outcome measures include the proportion of patients receiving albumin within 2 hours of diuretic, the proportion of patients receiving the full six doses of study treatment, the proportion of patients who receive open label 25% albumin, and the rate of recruitment. Physiologic, laboratory, and clinical data are collected until discharge from the ICU or until 30 days.

**Discussion:**

This is the first randomized trial to assess the use of hyperoncotic albumin in addition to diuretics in a general ICU population. Should this pilot study demonstrate feasibility, the primary outcome measure of the larger clinical trial will be the number of ventilator-free days, with secondary clinical outcome measures of duration of mechanical ventilation, length of ICU stay, episodes of hemodynamic instability and mortality. The addition of 25% albumin to standard diuretic therapy is a promising treatment in the post-resuscitation care of the critically ill patient.

**Trial registration:**

ClinicalTrials.gov NCT02055872; ISRCTN70191881.

## Background

### Study rationale

Many intensive care unit (ICU) patients require fluid resuscitation with crystalloids, colloids, or blood products in order to treat their critical illness. However, aggressive resuscitation can lead to fluid overload, characterized by peripheral and pulmonary edema, and has been associated with the development of acute respiratory distress syndrome (ARDS) and longer stays in the ICU, as well as higher mortality, compared with patients without fluid overload [1-3]. Observational data suggest that positive fluid balance is among the most important prognostic variables for ICU mortality [4], and a retrospective review of the use of intravenous fluids during the first four days of sepsis care in the VASST study showed that a more positive fluid balance at both 12 hours and day 4 correlated significantly with mortality [2]. In the ARDS population, positive fluid balance in addition to a high tidal volume ventilatory strategy is associated with worse outcomes [3], and randomized controlled trial data from Wheeler *et al.*[5] has shown a conservative intravenous fluid strategy to be of benefit with respect to improved lung function and duration of mechanical ventilation strategy in a broad population with ARDS. More recent data have suggested that in patients with acute lung injury complicating septic shock, adequate initial fluid resuscitation coupled with conservative late fluid management results in optimal outcomes [6]. Thus, in ICU patients without shock, maintenance of euvolemia and diuresis of excess fluid received during initial resuscitation may be beneficial, with loop diuretics such as furosemide being the standard therapy.

At the same time, critical illness and malnutrition can lead to hypoproteinemia, resulting in lower vascular oncotic pressure and a tendency for fluid to shift into the interstitial compartment, further worsening edema [7]. For such patients, a strategy of hyperoncotic colloid infusion followed by a diuretic such as furosemide makes physiologic sense, as the colloid promotes redistribution of fluid from edematous peripheral tissues back into the vascular compartment, where it can then be filtered and excreted by the kidneys. Research from our institution has confirmed that hyperoncotic albumin can improve colloid osmotic pressure in critically ill patients [8]. Furthermore, furosemide itself is heavily protein-bound, and in hypoproteinemic patients this results in an increased volume of distribution and lower concentrations of the diuretic in the loop of Henle [9]. The addition of albumin to furosemide has been shown to improve the volume of diuresis in several patient populations, including patients with renal failure [10-12], and cirrhosis [13].

Thus, in critically ill patients with hypoproteinemia and evidence of volume overload, there is a rationale for studying the addition of colloids to standard diuretic therapy. Despite this, there are few prospective studies, and only two randomized controlled trials, which have evaluated the addition of colloids to diuretics in critically ill patients (Table 1) [14-17]. Both randomized controlled trials evaluated the use of albumin in addition to furosemide. Makhoul and colleagues [18] randomized 30 mechanically ventilated patients with congestive heart failure to intravenous furosemide with or without 25% albumin, and did not demonstrate a difference in volume of diuresis obtained or serum chemistry data at 24 hours, although this may be due to the short (24-hour) course of treatment and the crossover study design. Furthermore, they did not report clinical outcomes [18]. Martin *et al.*[19] studied 40 mechanically ventilated patients with ARDS, and found that the addition of intravenous albumin to an infusion of furosemide, compared with furosemide infusion alone, is beneficial in patients with ARDS, resulting in improved volume of diuresis and improved oxygenation, as well as improved hemodynamic stability during diuresis [19]. In summary, there is promising, but inconclusive, evidence available to guide the use of albumin in addition to diuretics in a general ICU population.

**Table 1:** Studies evaluating 25% albumin vs. placebo for the diuresis of critically ill patients

**Reference**	**Type of study**	**Study population**	**Study intervention**	**Study outcome**
[14]	Non-randomized trial	Surgical ICU patients	Salt-poor albumin (*n* = 8) versus furosemide alone (*n* = 5); all patients received furosemide	Improved oxygenation as measured by ADO_2_ at 2 hours
[17]	Retrospective observational study	Medical ICU patients (*n* = 31)	Case patients received 25% albumin; control patients did not; all patients received furosemide infusion	No change in urine output or fluid balance
[18]	**Randomized controlled trial**	**Mechanically ventilated patients with congestive heart failure (*n*** **= 30)**	**250 mg frusemide diluted in 12.5 g albumin at a rate of 0.1 mg frusemide/kg/hour versus furosemide infusion alone**	**No difference in urine output or fluid balance at 24 hours**
[15]	Retrospective observational study	ICU patients	Cases patients (*n* = 30) received at least four doses of 100 ml 25% albumin; control patients (*n* = 25) received no albumin	No change in oxygenation, hemodynamics; higher positive fluid balance in cases
[19]	**Randomized controlled trial**	**Mechanically ventilated patients with ARDS (*n*** **= 40)**	**100 ml of 25% albumin every 8 hours versus placebo; furosemide infusion**	**Improved oxygenation, greater net negative fluid balance, better maintenance of hemodynamic stability**
[16]	Prospective matched case-control study	Mechanically ventilated patients with acute lung injury or ARDS	Case patients (*n* = 57) received 200 ml of 20% albumin and Lasix infusion; control patients (*n* = 57) received usual care	Lower net fluid balance, shorter ICU stay, reduced duration of mechanical ventilation; lower mortality

Randomized trials highlighted in bold text. ADO_2_, alveolar-arterial oxygen difference; ARDS, acute respiratory distress syndrome; ICU, intensive care unit.

The need for well-studied therapies to treat fluid overload in ICU patients therefore warrants a randomized controlled trial to evaluate the effectiveness of intravenous 25% albumin plus furosemide, compared with furosemide alone, in achieving improved total fluid balance, oxygenation, and hemodynamic stability at 72 hours, as well as improved ventilator-free days. Prior to embarking upon such a trial, we propose a pilot study to assess its feasibility.

### Study objectives

#### Primary objective

To determine the feasibility of conducting a randomized controlled trial in critically ill patients with hypoproteinemia and fluid overload, which will investigate the clinical effects of 25% albumin in addition to furosemide. Specific feasibility outcomes, and pre-specified criteria to determine feasibility, are outlined in Tables 2 and 3.

**Table 2:** Variables, measures, and methods of analysis

**Outcome**	**Hypothesis**	**Outcome measure**	**Covariates**	**Methods of analysis**
**Feasibility outcomes**				
Adherence to treatment	>85% adherence	Proportion receiving first dose of study treatment within 2 hours of furosemide	None	95% confidence interval of a proportion
Completion of treatment	>80% completion	Proportion receiving all six doses of study treatment	None	95% confidence interval of a proportion
Absence of hyperoncotic albumin in control arm	>85% in control group without 25% albumin	Proportion of patients assigned to control receiving no 25% albumin	None	95% confidence interval of a proportion
Randomization rate of eligible patients	>50% of eligible patients recruited	Proportion of eligible patients randomized	None	95% confidence interval of a proportion
Randomization rate of patients by clinical site	Average recruitment of one patient per site per week	Rate of recruitment per week	None	Average rate of recruitment
**Clinical outcomes**				
*Primary outcome*				
Duration of mechanical ventilation	Increased number of ventilator-free days in treatment group	Number of ventilator-free days	Duration of ventilation at time of randomization	*t* test, linear regression
*Secondary outcomes*				
Duration of mechanical ventilation	Decreased duration of mechanical ventilation in treatment group	Duration of mechanical ventilation (days)	Duration of ventilation at time of randomization	*t* test, linear regression
Episodes interrupting treatment with furosemide	Fewer episodes of interruption in treatment group	Number of episodes	None	*t* test
Need for dialysis	Smaller proportion of patients requiring dialysis in treatment group during ICU stay	Proportion of patients requiring dialysis during 30 days	None	Chi-squared or Fisher’s exact statistic
Length of ICU stay	Shorter length of stay in treatment group	Duration of ICU stay	Duration of ICU stay at time of randomization	*t* test, linear regression
ICU mortality	Decreased ICU mortality in treatment group during ICU stay	All-cause mortality (binary)	APACHE-2 and SOFA scores at randomization	Kaplan-Meyer survival curve (time to death)
30-day mortality	Decreased 30-day mortality in treatment group	All-cause mortality (binary)	APACHE-2 and SOFA scores at randomization	Kaplan-Meyer survival curve (time to death)
*Physiologic outcomes*				
Change in oxygenation	Greater increase PaO_2_/FiO_2_ ratio in treatment group at day 3 and day 5	Change in PaO_2_/FiO_2_ ratio	None	*t* test
	Greater decrease in oxygenation index in treatment group at day 3 and day 5	Change in oxygenation index	None	*t* test
Change in lung compliance	Greater increase in dynamic compliance in treatment group at day 3 and day 5	Change in dynamic compliance during study treatment (ml/cmH_2_O)	None	*t* test
Change in fluid balance	Greater net decrease in fluid balance in treatment group at day 3 and day 5	Change in net fluid balance (ml)	Net fluid balance at time of randomization	*t* test, linear regression
Change in body weight	Greater decrease in body weight in treatment group at day 3 and day 5	Change in body weight (kg)	Baseline weight	*t* test, linear regression
Urine output	Greater net urine output in treatment group at day 3 and day 5	Urine output during 3 days of study treatment (ml)	Net fluid balance at time of randomization	*t* test, linear regression
Dose of furosemide	Lower total amount of furosemide used in treatment group at day 3	Dose of furosemide during 3 days of study treatment (mg)	None	*t* test
Changes in serum albumin	Greater increase in serum albumin level in treatment group at day 3 and day 5	Change in serum albumin (g/l)	None	*t* test
Changes in colloid osmotic pressure	Greater increase in colloid osmotic pressure in treatment group at day 3 and day 5	Change in colloid osmotic pressure (mmHg)	None	*t* test
Changes in serum total protein	Greater increase in total protein in treatment group at day 3 and day 5	Change in total protein (g/l)	None	*t* test
Change in electrolytes	No difference between study groups for major electrolytes (sodium, potassium) at day 3 and day 5	Change in electrolytes between beginning and end of study treatment (mEq/l)	None	*t* test
**Subgroup analyses**				
ARDS	Improved oxygenation in patients with ARDS		None	Regression methods with appropriate interaction term
Severity of disease	Improved hemodynamic stability in patients with severe disease		None	Regression methods with appropriate interaction term
Time since recovery of hemodynamic stability	Improved hemodynamic stability in patients with recent hemodynamic instability	>48 hours versus <48 hours of hemodynamic stability (as defined by study eligibility criteria)	None	Regression methods with appropriate interaction term
**Sensitivity analyses**				
Per-protocol analysis		All outcomes	None	
Adjusting for baseline covariates		All outcomes	As described	Multivariable regression
Adjusted for dose of furosemide	Treatment with albumin results in improvement beyond that explained by dose of furosemide	All outcomes	Dose of furosemide	Multivariable regression

APACHE-2, Acute Physiology and Chronic Health Evaluation-2; ARDS, acute respiratory distress syndrome; FiO_2,_, fraction of inspired oxygen; ICU, intensive care unit; PaO_2_, partial pressure of oxygen in arterial blood; SOFA, Sepsis-related Organ Failure Assessment.

**Table 3:** Feasibility outcomes and estimated sample sizes

**Outcome**	**Demonstration of feasibility**	**Estimated actual proportion**	**Desired precision**	**Required sample size**
Administration of first dose of albumin within two hours of furosemide administration	Lower-bound of 95% confidence interval greater than 85%	0.925	0.075	47
Administration of at full 72 hours of study treatment	Lower-bound of 95% confidence interval greater than 80%	0.9	0.1	34
Absence of hyperoncotic albumin in control arm	Lower-bound of 95% confidence interval greater than 85%	0.95	0.1	18
Randomization rate of patients eligible by screening criteria	Lower-bound of 95% confidence interval greater than 50%	0.65	0.15	39

### Hypothesis

It is feasible to conduct a randomized controlled trial in critically ill patients with hypoproteinemia to investigate the clinical effects of 25% albumin in addition to furosemide.

### Secondary objectives

For this pilot study, we will assess feasibility outcomes, but also collect physiologic and clinical outcome data, including volume of diuresis, oxygenation, duration of ventilation, length of ICU stay, and mortality. However, we expect the pilot study to be underpowered to demonstrate changes in these outcomes. If the pilot study successfully demonstrates feasibility, these unblinded data will be incorporated in the planned larger trial, in a nested fashion. We hypothesize that the use of intravenous 25% albumin in addition to furosemide, as opposed to furosemide alone, increases the volume of diuresis achieved, improves oxygenation, prevents intravascular volume depletion at 72 hours, and results in more ventilator-free days.

## Methods/Design

### Study design

The FADE study is a single-center, parallel, pilot randomized controlled trial. The primary outcome is feasibility, with secondary physiologic and patient-important outcome data also being collected.

### Participants

The target population is one of critically ill adults with hypoalbuminemia who are in a recovery phase of critical illness and judged by the treating physician to be volume overloaded, based on clinical findings, such as peripheral edema, delayed weaning, or chest radiographic findings of interstitial or pleural fluid. Thus, inclusion criteria include: (1) admission to ICU; (2) age ≥ 18 years; (3) hemodynamic stability for at least 24 hours, defined as the absence of persistent (>1 hour) hypotension (systolic blood pressure < 90 mmHg) and tachycardia (heart rate >110), not currently on vasopressors, received less than 2 l crystalloid or colloid boluses or two units of packed red blood cells, maintenance fluids excluded; (4) hypoproteinemia, defined as serum albumin < 30 g/l, or total protein < 60 g/l; (5) clinical decision by the caregiving team to diurese at least 3 l of net fluid balance within the next 72 hours, for any reason.

Patients will be excluded for any of the following reasons: (1) known pregnancy; (2) patient or surrogate decision maker unable or unwilling to consent to blood product administration, including albumin; (3) acute kidney injury (RIFLE criteria ‘F’ or greater with either tripling of creatinine or creatinine >355 μmol/l (with a rise of >44) or average urine output below 0.3 ml/kg/hour for 24 hours) without any improvement in the previous 24 hours, or otherwise expected to necessitate dialysis within 48 hours in the opinion of the treating physician; (4) chronic kidney injury requiring dialysis; (5) clinically documented cirrhosis; (6) clinically documented nephrotic syndrome; (7) serum sodium levels greater than 150 mEq/l or serum potassium levels less than 2.5 mEq/l that cannot be treated prior to administration of study treatment; (8) inability to measure urine output and fluid balance accurately; (9) receipt of hyperoncotic albumin within preceding 24 hours; (10) previous enrollment in this trial, or any other research studies for which co-enrollment is not permitted; (11) estimated survival or ICU stay less than 72 hours.

### Recruitment plans and consent process

**Figure 1:**
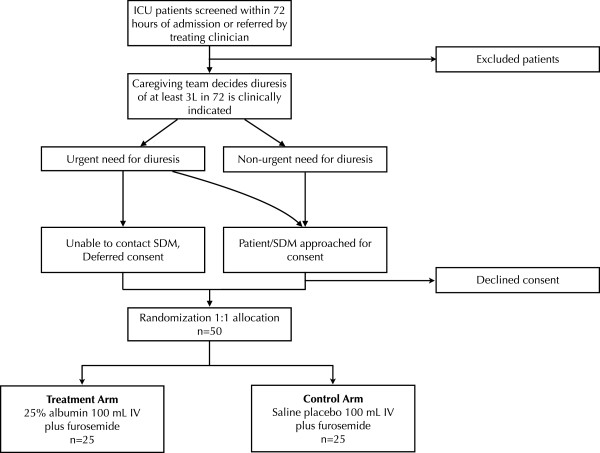
**Patient recruitment and randomization process.** ICU, intensive care unit; IV, intravenously, SDM, surrogate decision maker.

The study will be carried out at two different ICUs within a single tertiary care academic center (Hamilton General Hospital and the Juravinski Hospital and Cancer Centre, Hamilton, Ontario, Canada). Both sites are research-intensive and serve a high volume of general medical and surgical critically ill patients. The recruitment process is outlined in Figure 1 and Table 4. Patients will be screened by study investigators within 72 hours of admission to the ICU. Patients present in the ICU for longer than 72 hours can be referred to study investigators by the team providing care if they believe that the patient might qualify for the study. Patients or surrogate decision makers will be approached by the research team and informed of the study rationale and methods, and provided with literature about the study. They will then be given an opportunity to consider study enrollment, in person or via phone. If the patient is willing, consent for the research study as well as blood product administration, if it has not already been received by the caregiving team, will be obtained. Patients will be screened and randomized once the caregiving team decides diuresis is necessary, but prior to administering diuretics from the caregiving team.

**Table 4:** Schedule for enrollment, interventions, and assessments

	**Study period**							
	**Enrollment**	**Allocation**	**Post-allocation**					**Close-out**
**Time point**	**Day -1**	**0**	**Day 1**	**Day 2**	**Day 3**	**Day 4**	**Day 5**	**Day 30**
**Enrollment**								
Eligibility screen	×							
Deferred or informed consent	×							
Allocation		×						
**Interventions**								
Treatment arm			×/×	×/×	×/×			
Control arm			×/×	×/×	×/×			
**Assessments**								
*Baseline variables*								
Demographics	×							
Past medical history	×							
Type of diagnosis on admission	×							
APACHE-2 score at randomization	×							
SOFA score at randomization	×							
Diagnosis of ARDS at randomization	×							
Time since hemodynamic stability	×							
Length of ICU stay at randomization	×							
*Laboratory results*								
Renal function tests			×	×	×	×	×	
Serum chemistry			×	×	×	×	×	
Serum albumin levels			×	×	×	×	×	
Serum total protein levels			×		×		×	
Colloid osmotic pressure			×		×		×	
Blood gas analysis			×	×	×	×	×	
Troponin I levels			×	×	×	×	×	
Serum lactate levels			×	×	×	×	×	
Complete blood count			×	×	×	×	×	
*Physiologic data*								
Vital signs			×	×	×	×	×	
Glasgow Coma Scale rating			×	×	×	×	×	
Weight			×	×	×	×	×	
Ventilator data			×	×	×	×	×	
Fluid intake and output			×	×	×	×	×	
Diuretic doses and timing			×	×	×	×	×	
*Clinical outcomes*								
Study treatment held?			×	×	×	×	×	
Need for dialysis?			×	×	×	×	×	×
Hypotension requiring fluids			×	×	×	×	×	
Hypotension requiring vasopressors			×	×	×	×	×	
Suspected infection?			×	×	×	×	×	
Intubation status			×	×	×	×	×	×
Date of transfer from ICU			×	×	×	×	×	×
Date of death			×	×	×	×	×	×

APACHE-2, Acute Physiology and Chronic Health Evaluation-2; ARDS, acute respiratory distress syndrome; ICU, intensive care unit; SOFA, Sepsis-related Organ Failure Assessment.

Alternatively, if a treating physician believes that a patient might qualify for the study, and is in urgent need of diuresis, the patient can be considered for deferred consent. To be eligible for deferred consent, patients or surrogate decision makers must have already given consent for administration of blood products. Patients will then be randomized to a study arm and diuretics administered. Study investigators will try to obtain consent from the surrogate decision maker within the next 48 hours. If the surrogate decision maker cannot be located, the patient will be withdrawn from the study. Similarly, if the surrogate decision maker declines participation in the study, the participant will be withdrawn from the study. The deferred consent process is only to be used if the need for diuresis is urgent, and the surrogate decision maker cannot be reached in a timely fashion.

Many strategies have been implemented to aid in recruitment for this pilot study, including the use of recruitment posters and presentations to familiarize ICU staff in participating centers with the study aims and methods. All investigators are either critical care physicians or fellows at the two ICUs where the study is taking place and will aid in recruitment. The inclusive eligibility criteria should also facilitate timely completion of the study.

### Randomization

The randomization process is outlined in Figure 1. Participants will be randomly assigned to either the control or experimental group in a 1:1 fashion with allocation concealment using an online computer generated randomization schedule. Randomization will be done at the time that the treating physician judges diuresis to be necessary. Allocation of patients to the study group will be carried out by the transfusion medicine service, who will contact a web-based randomization system set up by the statistics team. The transfusion medicine personnel responsible for assignment are distinct from the study personnel enrolling subjects in the study in the ICUs. The transfusion medicine service will keep an electronic list of patient names, study ID numbers, and treatment allocation. Study investigators will not have access to this list and will have to contact the transfusion medicine service to unblind patients for any reason, for example, suspected adverse reaction to a study treatment.

### Blinding

Trial participants, care providers, data collectors, and outcome assessors will be blinded to treatment assignment. Saline placebos will be produced by the hospital’s pharmacy service under sterile conditions by instilling 100 ml of normal saline into evacuated glass bottles identical to those used by the manufacturers of the 25% albumin. The placebos will then be stored in the transfusion medicine department for use in the study. Once a patient has been randomized and assigned to a treatment group, the transfusion medicine service will cover the assigned treatment bottles with an opaque plastic sleeve and dispense them to the ICU, along with opaque intravenous tubing. Fluids will be infused into direct intravenous ports rather than distal intravenous tubing so that all study treatments will be administered directly from the covered bottles and opaque tubing without intervening clear intravenous tubing, which may threaten blinding. The opaque sleeves and intravenous tubing will allow bedside nurses to infuse the fluids without compromising blinding of themselves or the caregiving physicians. To further reduce bias of reporting, the majority of the outcomes of interest in the study are objective (that is, laboratory values, physiologic measurements), rather than subjective outcomes requiring adjudication.

To ensure the safety of study participants, a protocol for emergency unblinding exists. In the event of an adverse reaction that could be attributed to the study treatment (for example, anaphylactic reaction or transfusion-related reaction), the study coordinators will be notified and will unmask group assignment by communicating with the third party personnel in the blood bank responsible for group allocation. Code breaks will occur only under exceptional circumstances. All code breaks (with justification) will be reported on the relevant case report forms.

### Interventions

**Figure 2:**
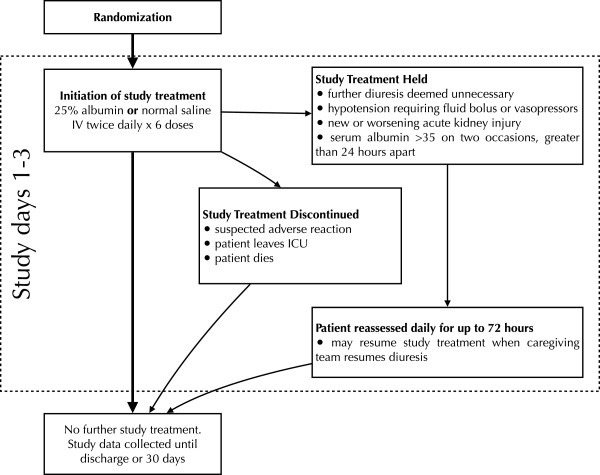
**Patient treatment and data collection protocol.** IV, intravenous.

The treatment and data collection protocol is outlined in Figure 2. The placebo will be 100 ml of normal (0.9%) saline, administered as an intravenous infusion twice daily. This volume is negligible compared with the total fluid intake of critically ill patients (for feeds, medications, and so on), and unlikely to adversely affect study or patient outcomes, but will still provide adequate blinding of caregivers and study personnel.

The study treatment, either 100 ml of 25% albumin or 100 ml of saline placebo, will be administered as an infusion over 60 minutes, within 2 hours of the patient’s prescribed dose of furosemide. Clinicians will be encouraged to prescribe furosemide twice daily so as to facilitate the timely administration of study treatment prior to furosemide administration.

In the event of new or worsening acute kidney injury, hypotension requiring further boluses of intravenous fluids or vasopressor agents, or if the caregiving team decides that further diuresis is unnecessary, study treatment will be held as long as the caregiving team also holds the furosemide. If a patient’s serum albumin level remains greater than 35 g/l for two measurements greater than 24 hours apart, study treatment will likewise be held. Patients will be monitored for up to 72 hours from randomization and those patients who the caregiving team decide need further diuresis, and have serum albumin levels <35 g/l, will resume the study treatment to which they were assigned.

If the participant develops a suspected adverse reaction to either the albumin, placebo, or furosemide, or the patient leaves the ICU (is transferred to the ward, another institution, or dies) study treatment will be discontinued.

No further administration of colloid intravenous fluids (albumin or starch) will be permitted while the patient is receiving study treatment. In the event that blood product transfusion is required for low serum hemoglobin levels (as opposed to active bleeding with hemodynamic instability), one dose of the study treatment may be held and the prescribed furosemide may be given after the blood transfusion is completed, at the discretion of the caregiving team.

Nursing staff will be provided with a bedside schedule to ensure that the study drug is administered in a timely fashion and that necessary measurements are performed and blood samples collected. The short duration of treatment and the ease of study drug administration will facilitate adherence across both ICUs.

### Outcomes

Outcome measures are detailed in Table 2. Our primary outcome in this pilot randomized controlled trial is feasibility, which will be assessed using five major feasibility outcomes: (1) greater than 85% adherence to protocol for randomized patients, defined as the administration of at least the first dose of study treatment (albumin or placebo) within two hours of the first administration of furosemide. This measure was chosen based on the half-life of serum albumin to increase serum colloid osmotic pressure [8]; (2) more than 80% of randomized patients receiving the full six doses of treatment study drug; (3) fewer than 15% of patients receiving non-protocolized hyperoncotic albumin administration; (4) recruitment of more than 50% of patients eligible by screening criteria; and (5) randomization rate of at least one patients by clinical site per week.

If all five criteria are met, feasibility will have been determined. Likewise, if the majority (more than three of five) of the criteria are met, and the unmet criteria could potentially be addressed with reasonable changes to the study protocol, feasibility will have been determined. If the unmet criteria cannot be addressed with changes to study protocol, or only a minority (one or two of five) of criteria are met, then non-feasibility will have been determined.

We will also collect clinical and physiologic outcome data, in anticipation of conducting a larger follow-up trial, should this study determine feasibility. The primary outcome for that trial will be the number of ventilator-free days from time of randomization. Ventilator-free days are defined over a 28-day period, with a patient receiving one ventilator-free day for each 24 hour period spent without the need for mechanical ventilation. Patients who die during the 28 days will automatically be assigned 0 ventilator-free days [20]. This is a commonly used outcome in critical care trials, as it incorporates patient-important outcomes of duration of mechanical ventilation and mortality, both of which have been associated with volume overload in observational studies.

The secondary clinical outcomes that we will collect data for include: (1) duration of mechanical ventilation, measured from time of randomization until extubation that is successful for 24 hours or more, recorded in days; (2) number of episodes of interrupting treatment with furosemide (for example, hypotensive episodes, renal failure, contraction alkalosis); (3) proportion of patients receiving dialysis; (4) length of ICU stay from randomization; (5) ICU mortality; and (6) 30 day mortality. We hypothesize that the addition of 25% albumin will lead to fewer interruptions of diuresis, a reduced incidence of acute kidney injury, and a shorter stay in the ICU.

The secondary physiologic outcomes that we will collect data for include: (1) changes in oxygenation (FiO_2_, PaO_2_/FiO_2_ ratio, oxygenation index) from baseline at day 3 and day 5; (2) changes in dynamic lung compliance, reflecting changes in extravascular lung water, at day 3 and day 5; (3) changes in total fluid balance and body weight, and amount of urine output from baseline at day 3 and day 5; (4) total dose of furosemide used, to assess whether study treatment improves the physiologic response to furosemide; (5) changes in serum albumin and colloid osmotic pressure between treatment groups from baseline at day 3 and day 5, to ensure that the experimental treatment results in the expected increases in these measurements; (6) changes in electrolyte levels (potassium level less than 2.5 mEq/l, sodium level >150 mEq/l), as a way to ensure that no major, potentially harmful changes in serum electrolytes occur as a result of study treatment.

### Sample size calculations

We determined that the greatest threat to the feasibility is the ability to administer the first dose of study treatment in a timely fashion, within two hours of the first administration of furosemide. As such, it was selected as the outcome used to determine our sample size. Assuming an actual adherence of 92.5%, to demonstrate at least an 85% adherence, a sample size of 47 is required. Other important feasibility outcomes include the administration of 72 hours of study treatment to each patient, and the absence of hyperoncotic albumin administration in the control arm. Estimated sample sizes required to show those outcomes with the desired level of precision are shown in Table 3.

### Data collection

Data to be collected are listed in Table 4. Demographic information, prior medical conditions, and reason for admission to ICU will be recorded. The Acute Physiology and Chronic Health Evaluation (APACHE), and Sepsis-related Organ Failure Assessment (SOFA) will be used at the time of ICU admission, the time of randomization, and the treatment end. The administration and doses of intravenous fluids, blood products, and diuretics will also be recorded. Data related to rationale for diuresis will also be collected (peripheral edema, pulmonary edema on chest X-ray, low urine output, elevated central venous pressures or left atrial pressures). Study outcome data collected daily will include vital signs and hemodynamics (if central venous monitoring or pulmonary artery catheters are in place), fluid balance, hourly urine output, weight, ventilator settings (if patient is ventilated), intubation status (including reasons for intubation or re-intubation), oxygenation parameters (SaO_2_, FiO_2_, PaO_2_/FiO_2_ ratio, oxygenation index). Blood samples, to determine standard and extended electrolyte levels, renal profile, complete blood count with differential, serum albumin and total protein levels, and blood gas data, are routinely collected in patients undergoing diuresis and will be collected at least daily. Colloid osmotic pressure and serum total protein will also be assessed at baseline, day 3 and day 5. Study data will be collected from the time of ICU admission to hospital discharge or 30 days after enrollment. All of these data are measured routinely in the ICU and subject to minimal interobserver variation. Colloid osmotic pressure will be measured using the Wescor model 4220, which has a precision of ± 0.03 mmHg [21].

Subject data will be collected daily through examination of the patient chart, including electronic ICU charting and the Meditech laboratory reporting system. Standardized electronic case report forms will be used to collect patient and study data. Data recording will be performed using standardized case report forms in duplicate to ensure minimal human error. Data from case report forms will be entered into an electronic database on a secure computer server. Given the short time of study treatment (3 days) and intensive follow-up to 5 days, retention of study subjects is not anticipated to pose a problem. Similarly, longer-term follow-up data for our outcomes of interest (transfer from ICU, ventilator-free days, and death) are easily retrieved from hospital records.

### Data management

Data will be entered using electronic case report forms into a computerized database stored on a secure server in the Department of Epidemiology and Biostatistics at McMaster University. All collected data will be kept under secure conditions in keeping with Good Clinical Practice and McMaster University’s research ethics board guidelines for a minimum of 7 years following study completion.

### Statistical methods

All *P* values will be two sided, with a level of *P* ≤ 0.05 indicating statistical significance. All data will be analyzed using statistical software on an intention-to-treat basis. All randomized participants, regardless of protocol adherence, will be included in the main analysis.

Feasibility outcomes will be calculated as proportions with 95% confidence intervals, with feasibility being met if the lower-bound of the 95% confidence interval is above the level pre-specified for feasibility (Table 3). Data regarding our primary and secondary clinical outcomes will also be collected during this pilot study for future analysis; however, no analysis of clinical outcomes will be made for this pilot study, as the main purpose is to inform the definitive trial. Data collected from this pilot study will remain blinded and eventually incorporated into the dataset for the definitive trial, with the following pre-specified analyses.

Comparisons between the two treatment groups at a single time point (for example, baseline variables, number of patients requiring fluid bolus or vasopressors for hypotension, total dose of furosemide, and so on) will made using unpaired Student’s *t* test. Comparisons within a treatment group at different time points (change in serum albumin, colloid osmotic pressure, total protein, weight, fluid balance, oxygenation, lung compliance at day 3 and day 5, and so on) will be analyzed in a similar fashion. Multiple between-groups comparisons of continuous variables will be analyzed by repeated-measures analysis of variance. Linear regression will be used where appropriate to correct for baseline variables. Ventilator-free days will be defined as the number of 24-hour periods in which a patient does not need ventilation during 28 days (patients who die, by definition, will have 0 ventilator-free days). Survival analysis will be performed to assess 30-day mortality.

*A-priori* analyses include stratifying patients by illness severity at time of randomization (using APACHE and SOFA scores), presence of ARDS, patients admitted with sepsis, patients admitted with congestive heart failure, and time since recovery of hemodynamic stability (greater than or less than 48 hours from discontinuation of vasopressors). All subgroups carry biological plausibility for differing effects of the intervention, with the hypothesis of greater improvement in those patients with more severe illness, presence of ARDS, and shorter time since hemodynamic stability.

### Data monitoring and auditing

Study participants will be closely monitored in the ICU. Suspected harms of the study treatment will be reported directly to study investigators. Case report forms will also be used to record any suspected harms and clinically important differences between treatment and control groups, including patient mortality, length of ICU stay, ventilator-free days, and electrolyte abnormalities, which will be analyzed using the statistical methods outlined in Table 2. Data monitoring and quality assurance of the study will be undertaken by the investigators. The local investigator (CH) will undertake responsibility for such duties.

Study participants may be withdrawn from the study early if there is suspicion of treatment harm by the team caring for the participant, such as an allergic reaction, worsening renal function, or worsening hypernatremia; or if consent for study therapy is withdrawn by the participant or surrogate decision maker at any time. Treating physicians cannot withdraw patients from the study, but can decide to withhold study treatment. Participants who withdraw consent to the study will be asked whether they are withdrawing from any or all of: (1) study treatment, (2) future data collection, or (3) primary outcome data collection.

Formal data monitoring will not be performed in this small pilot randomized controlled trial, owing to both its small sample size and the small risk the intervention poses to study participants. A formal data-monitoring committee will be created to oversee the larger follow-up trial, should this pilot study demonstrate feasibility.

### Research ethics approval and confidentiality

The study protocol, written and verbal consent forms, data to be collected, and recruitment posters have been approved by the Hamilton Integrated Research Ethics Board (Project #14-002). Any modifications of the protocol that might impact patient safety, confidentiality, or other significant conduct of the study will be reviewed by the Hamilton Integrated Research Ethics Board before implementation. Minor administrative changes will be reviewed by the investigative team but not the Hamilton Integrated Research Ethics Board.

All study information will be stored securely in locked cabinets or on secure, password-protected computer systems with limited access at the study sites. All case report form data, laboratory specimens, and administration forms will be identified with a coded identification number to maintain patient confidentiality. Records containing patient names and other identifying information will be stored in a separate locked area with limited access. No patient-identifying information will be published or released to anyone outside of the study team.

### Access to data

Data management will be performed by members of the Department of Epidemiology and Biostatistics at McMaster University. The study investigators will have full access to study datasets. External requests for study data will be granted, however any information that is shared will be blinded to any identifying participant information.

### Ancillary and post-trial care

Patients enrolled into the study will receive routine care from the intensive care team following completion of study treatment. Should this study, or its follow-up studies, provide evidence of the effectiveness, no further provision of treatment to participants will be required, as the study treatment is for short-term use only.

### Dissemination policy

There are no stipulations on publication in place by any party, including the trial sponsors. The study investigators will publish the results of the pilot study, including feasibility outcomes and characteristics of the study cohort, should feasibility be demonstrated. Outcomes analyzed by the treatment group will only be published if non-feasibility is demonstrated; otherwise pilot outcome data will be retained for incorporation in the larger follow-up study. Attempts will be made to accomplish publication within 6 months of trial completion, and in keeping with the CONSORT guidelines for randomized controlled trial publication [22]. Authorship for publications will be in keeping with guidelines from the International Council of Medical Journal Editors [23]. Following the completion of this pilot study, and the larger, follow-up study (should this pilot demonstrate feasibility) a completely de-identified data set will be archived for sharing purposes.

## Discussion

The FADE study is the first prospective randomized controlled trial to assess the use of hyperoncotic albumin in addition to diuretics in a general ICU population. Should this pilot study demonstrate feasibility, the primary outcome of the larger clinical trial will be an increase in the number of ventilator-free days, with secondary clinical outcomes of reductions in the duration of mechanical ventilation, length of ICU stay, episodes of hemodynamic instability, and mortality. The addition of 25% albumin to standard diuretic therapy is a promising treatment in the post-resuscitation care of critically ill patients with edema.

## Trial status

The trial is not yet recruiting.

## Abbreviations

APACHE: Acute physiology and chronic health evaluation; ARDS: Acute respiratory distress syndrome; CONSORT: Consolidated standards of reporting trials; FiO_2_: Fraction of inspired oxygen; ICU: Intensive care unit; PaO_2_: Partial pressure of oxygen in arterial blood; RIFLE: ‘Risk, injury, failure, loss, end-stage renal disease’; SaO_2_: Oxygen saturation; SOFA: Sepsis-related organ failure assessment.

## Competing interests

The authors declare that they have no conflicts or competing interests.

## Authors’ contributions

SJWO and IM conceived and designed the study, wrote the study protocol and grant applications, will be involved in study recruitment, and wrote and approved the final manuscript. MM designed the study, reviewed the study protocol, and critically revised and approved the final manuscript. CH conceived and designed study, reviewed the study protocol, will be involved in study recruitment, and critically revised and approved the final manuscript. All authors read and approved the final manuscript.
